# Landscape modeling as a surveillance strategy for *Trypanosoma cruzi* vectors in Brazil

**DOI:** 10.1186/s13071-026-07481-3

**Published:** 2026-06-03

**Authors:** Felipe de Oliveira, Raphael Testai, Matheus Pinheiro Ferreira, Cleber Galvão, Diogo Souza Bezerra Rocha, Ana Maria Jansen, Samanta Cristina das Chagas Xavier

**Affiliations:** 1 Laboratory of Tripanosomatid Biology, Oswaldo Cruz Institute, Oswaldo Cruz Foundation (FIOCRUZ), Rio de Janeiro, RJ Brazil; 2 The Graduate Program in Computational and Systems Biology of the Instituto Oswaldo Cruz (PGBCS/IOC/Fiocruz), Rio de Janeiro, RJ Brazil; 3 Department of Forest Sciences, “Luiz de Queiroz” College of Agriculture, University of São Paulo (USP/ESALQ), Piracicaba, SP Brazil; 4 National and International Reference Laboratory in Triatomine Taxonomy, Oswaldo Cruz Institute, Oswaldo Cruz Foundation (FIOCRUZ), Rio de Janeiro, RJ Brazil; 5 International Institute for Sustainability, (IIS), Rio de Janeiro, RJ Brazil

**Keywords:** Ecological niche models, Land use and land cover, Triatomines, *Trypanosoma cruzi*, Risk of transmission

## Abstract

**Background:**

The transmission of *Trypanosoma cruzi* in nature is closely linked to the ecology of its vectors, the triatomine insects. Although the geographic distribution of these species is relatively well-known, understanding the characteristics that define their ecological niche and how human interventions alter these conditions remains limited. Rapid and intense environmental changes, driven by activities such as agricultural expansion, raise important questions about how vector ecology is being transformed and the new implications for Chagas disease transmission risk.

**Methods:**

This study aimed to develop Ecological Niche Models (ENMs) to map the environmental suitability for 14 triatomine species and *T. cruzi* in Brazil and to evaluate the impact of landscape modifications. Environmental variables were selected based on Spearman correlation, and models were generated using Maxnet, Random Forest, and Support Vector Machines. To ensure prediction robustness, only models with a True Skill Statistic (TSS) ≥ 0.7 were considered. Based on these predictions, a risk classification was developed that integrates vector diversity and parasite presence to identify areas with higher transmission risk.

**Results:**

The results revealed a significant ecological transition of the vectors, which are adapting from natural ecosystems to agroecosystems, particularly pastures and soybean cultivation, bringing the transmission cycle closer to humans. Risk analyses indicated that the Midwestern, Northern, Northeastern, and Southeastern regions are the most vulnerable, with actual risk strongly associated with modified landscapes.

**Conclusions:**

The integrated use of geotechnologies proved essential for identifying and understanding the spatial distribution of vectors and the parasite, as well as for quantifying the impact of human activity. These findings provide important insights for improving Chagas disease surveillance and control strategies, considering the environmental complexity and ecological heterogeneity involved in its transmission.Graphical abstract is mandatory for publication in this journal. Please provide the graphical abstract.The graphical abstract has been included as a attachment

**Graphical Abstract:**

**Figure:**
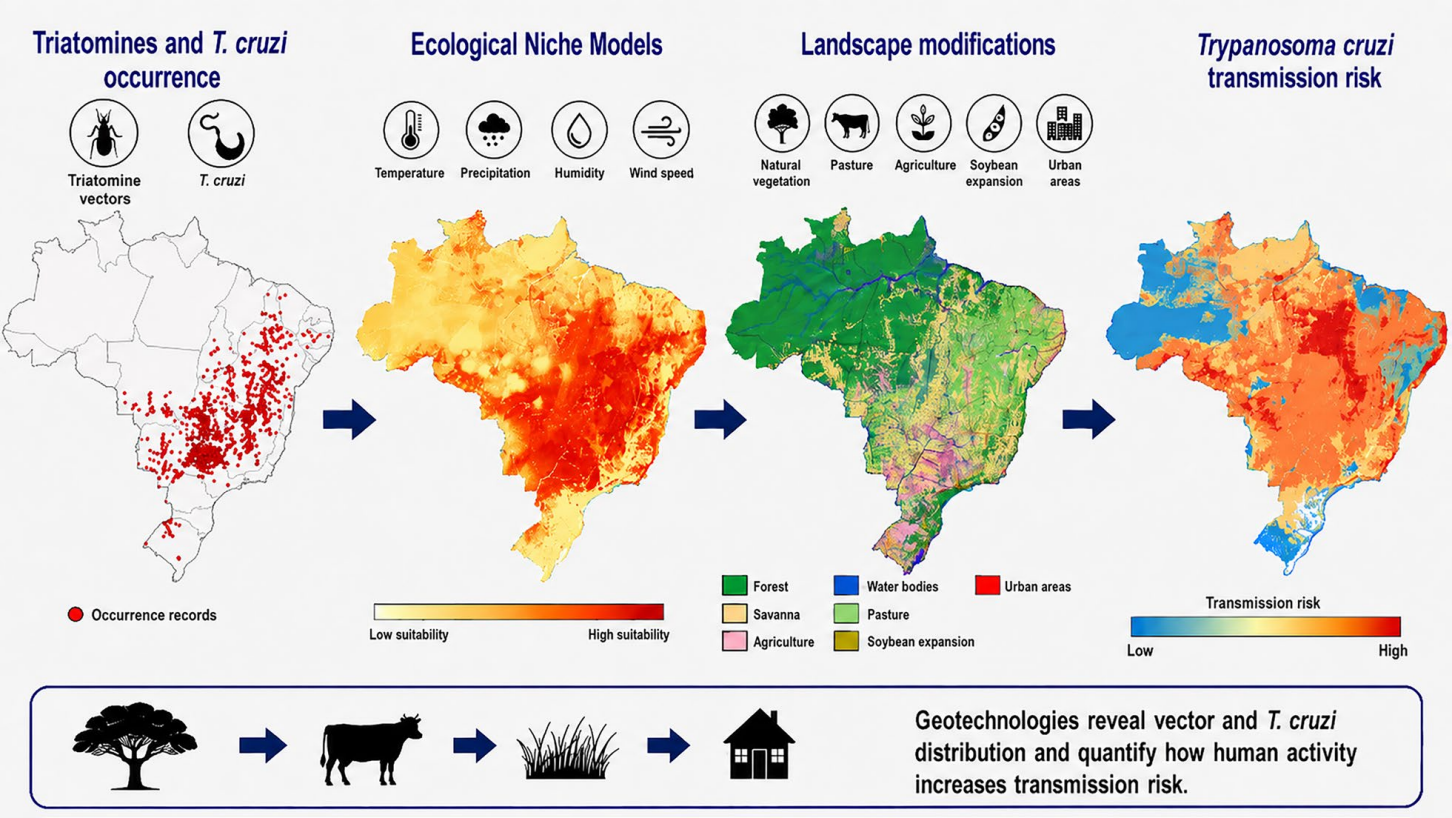

**Supplementary Information:**

The online version contains supplementary material available at 10.1186/s13071-026-07481-3.

## Background

*Trypanosoma cruzi* [1] is a flagellated protozoan belonging to the Trypanosomatidae family; it is widely distributed in nature, occurring in all Brazilian biomes. This parasite is characterized by extreme genetic, biochemical, and molecular diversity, which partly explains its significant biological plasticity, a fact that remains an open question to date. This taxon can be defined as multi-host since it infects all tissues of hundreds of mammalian species in nature [2] and is transmitted by hemipteran insects of the subfamily Triatominae (Hemiptera: Reduviidae). This subfamily currently comprises 158 species, including 155 extant and three extinct, distributed among 18 genera and five tribes, of which 64 are known from Brazil [3]. Currently, seven genotypes or DTUs (discrete typing units) are recognized in the different genetic lineages of *T. cruzi*: TcI–TcVI and TcBat [4].

In humans, infection with *T. cruzi* is called Chagas disease (CD), which has a complex epidemiological profile. In Brazil, CD is considered reemerging, with cases most commonly occurring as outbreaks, mainly resulting from ingestion of food contaminated with the feces of infected triatomine bugs, especially in the Amazon region [5].

American trypanosomiasis, primarily an enzootic disease caused by *T. cruzi*, affects numerous species of mammals and triatomines. These cycles can result in human infection under different circumstances. Understanding the peculiarities of the local landscape ecology and the biotic and abiotic variables that modulate this complex transmission system is essential for defining and establishing prophylaxis and control measures for humans [6].

In the current epidemiological profile, understanding the transmission cycles of *T. cruzi* in the wild is essential, given that it is a typically wild enzootic disease and human infections result from contact with wild triatomines. This contact results from human exposure directly in the wild environment or in the domestic/peridomestic environment because of the invasion of wild vectors [7]. In this context, data on the distribution of insect vectors and wild and domestic hosts are used in combination with environmental, anthropogenic, demographic, and socioeconomic factors to model the risk of human infection [8].

The Southern Cone Initiative, launched in 1991, successfully reduced Chagas disease transmission through *Triatoma infestans* control and blood bank screening, leading to Brazil's certification as free of transmission by this vector in 2006 [9]. However, considering vectorial transmission interrupted solely based on the control and/or elimination of a single triatomine species is quite risky. Therefore, to prevent new outbreaks of oral or vector-borne contamination, it is essential to adopt new approaches to analyzing environments at risk of invasion by wild vector species.

Brazil, with its vast territory and diverse biomes, supports a high diversity of triatomines [10]. Although the geographic distribution of triatomine species is widely documented in the literature [11], landscape features and potential environmental changes that influence the ecology of these vectors still require further investigation, thereby providing a basis for predicting the potential expansion or contraction of triatomine populations in response to future changes [12]. This study explores changes in landscapes and triatomine-related risk scenarios in Brazil over the past 4 decades (1985–2023). By identifying areas of environmental suitability for triatomine species across land use and land cover classes, the study identifies patterns of landscape change, highlights areas of vector presence in anthropic environments, and discusses the implications of these alterations for the risk of Chagas disease transmission. Although domiciliary transmission of Chagas disease has been significantly reduced, the disease's reemergence in several regions underscores the urgent need to investigate the enzootic cycles of *T. cruzi* nationwide.

## Methods

### Acquisition of climatic and environmental variables

The climatic variables were obtained from the WorldClim image database (https://www.worldclim.org/) at a resolution of 30 arcseconds (~ 1 km^2^); 19 bioclimatic variables, all in raster format (GeoTIFF), were acquired. The variables wind speed and water vapor pressure are provided in WorldClim with monthly values. Therefore, the images were unified to analyze the range between annual maximum and minimum values.

The landscape variables were obtained from the Google Earth Engine (GEE) platform (https://earthengine.google.com/). The variables used included the Normalized Difference Vegetation Index (NDVI) and Enhanced Vegetation Index (EVI), derived from the MODIS sensor onboard the Terra satellite, using the dataset (MODIS/061/MOD13A2) at a 1000 m resolution, in raster format (GeoTIFF). Land Surface Temperature (LST) was obtained from the dataset (MOD11A1 V6.1) with a spatial resolution of 1000 m, and Topographic Diversity was derived from the dataset (CSP/ERGo/1_0/Global/SRTM_topoDiversity) from the Shuttle Radar Topography Mission (SRTM). All images were resampled to 30 arcseconds (~ 1 km^2^). The NDVI, EVI, and LST variables were derived by taking the median pixel values across images from 2000 to 2022.

The topographic variables were obtained from the AMBDATA database (http://www.dpi.inpe.br/Ambdata/index.php), which is produced by the National Institute for Space Research (INPE). The variables used included altitude, slope, and aspect, generated from Shuttle Radar Topographic Mission (SRTM) data. All images were resampled to a spatial resolution of 30 arcseconds (~ 1 km^2^). The selection of variables for Ecological Niche Modeling (ENM) was performed using Spearman's correlation among them, with variables selected that had a correlation coefficient within the range of − 0.6 ≤ *α* ≤ 0.6.

### Acquisition of database of occurrences

To identify the environmental factors influencing the distribution of *T. cruzi* vectors in Brazil, this study selected 14 triatomine species based on their abundance in different biomes. Occurrence data were compiled from several databases, including DataTri [13], SpeciesLink (http://www.splink.org.br/), and the Global Biodiversity Information Facility (GBIF, https://www.gbif.org/). Additionally, *T. cruzi* occurrence data were obtained from the Trypanosoma Collection of Wild, Domestic Mammals, and Vectors (COLTRYP) database (http://coltryp.fiocruz.br/). This approach enabled us to investigate the distinct environmental characteristics associated with the ecological niches of these species and cover the diversity of habitats occupied by these vectors. After compilation, we performed a rigorous data filtering process to remove duplicate records, records with imprecise or inconsistent coordinates, records located outside the study area, records assigned to municipality centroids, and records associated with biological collections or institutions when these coordinates did not correspond to the actual collection site. In addition, spatial filtering was applied to reduce spatial autocorrelation among occurrence records by retaining only one record per 1-km pixel, avoiding duplicate occurrences within the same spatial unit used in the modeling process. After this filtering procedure, the final number of occurrence records retained for each species is presented in Table 1.

**Table 1:** Number of occurrence points of triatomine species and *Trypanosoma cruzi*, before and after the cleaning and spatial filters carried out in the rstudio software

Triatomines	Occurrences	Filtered
*Panstrongylus megistus*	4677	2205
*Panstrongylus geniculatus*	1210	313
*Panstrongylus lutzi*	812	483
*Panstrongylus tibiamaculata*	161	67
*Triatoma pseudomaculata*	3421	1206
*Triatoma sordida*	1591	978
*Triatoma brasiliensis*	3307	859
*Triatoma rubrovaria*	398	105
*Triatoma vitticeps*	818	153
*Triatoma melanocephala*	254	110
*Rhodnius neglectus*	736	168
*Rhodnius pictipes*	188	78
*Rhodnius robustus*	279	80
*Rhodnius nasutus*	608	87
*Trypanosoma cruzi*	678	148

### Ecological niche modeling

The modleR package in R was used for the ecological niche models. These models were developed by the research team at the Botanical Garden of Rio de Janeiro. Three machine learning algorithms were employed: Maxent, Random Forests, and Support Vector Machines. Multiple algorithms were used to reduce algorithm-specific bias and increase prediction robustness, as each method may capture different aspects of the relationship between species occurrence and environmental predictors. To test the models' accuracy, the database was divided into five partitions: 20% of the occurrence points were used to evaluate the models' performance, and the remaining 80% were used to train the algorithms. The k-fold cross-validation methodology was applied with ten iterations for each algorithm. The selection of models with good performance was based on their TSS (True Skill Statistics) scores, with a threshold of TSS ≥ 0.7. The final suitability model for each species was generated using an ensemble approach that combined the outputs of the three algorithms used in this study: Maxent, Random Forests, and Support Vector Machines.

### Landscape analysis of suitable areas

The landscape characteristics of the species' suitable areas were analyzed using an ensemble model. To identify the landscape features, the suitable areas were isolated and converted into raster files with suitability polygons. Then, the Google Earth Engine (GEE) platform was used to obtain information for the suitability polygons, using the land use and land cover dataset from MapBiomas (https://brasil.mapbiomas.org/) from Collection 9, covering the period from 1985 to 2023.

### Risk analysis

Based on the environmental suitability of vectors and the parasite, we used an ecological niche modeling approach to identify areas with varying transmission risk for *T. cruzi*. This approach was applied to 14 vector triatomine species and the parasite itself (*T. cruzi*). A combined spatial analysis was then developed from the resulting binary predictions of the distribution models (presence = 1; absence = 0) to integrate the information and generate a risk classification.

Fifteen binary raster maps in GeoTIFF format were used, corresponding to the potentially suitable areas for each triatomine species and for *T. cruzi*. The binary maps of the 14 triatomine species were summed pixel by pixel, resulting in a vector diversity raster, whose value represents the number of species with suitability for each cell (ranging from 0 to 14). The *T. cruzi* binary map was used to indicate areas with a current transmission risk. Pixels with a value of 1 were considered as areas with the parasite's presence. Based on the combination of vector abundance and the presence/absence of *T. cruzi*, the areas were categorized into seven risk classes, according to the criteria described.

The final risk map was classified into six classes by combining vector species richness and the occurrence of *Trypanosoma cruzi*. Classes 1 to 3 correspond to potential risk areas, characterized by the presence of vector species but without records of *T. cruzi*. These classes were graded according to vector species richness: Class 1: low potential risk (1–4 species), Class 2: medium potential risk (5–9 species), and Class 3: high potential risk (≥ 10 species). Classes 4 to 6 correspond to real risk areas, where vector species and *T. cruzi* records co-occur. These areas were also classified by vector species richness: Class 4: low risk (1–4 species), Class 5: medium risk (5–9 species), and Class 6: high risk (≥ 10 species).

## Results

### Selection of variables

**Fig. 1:**
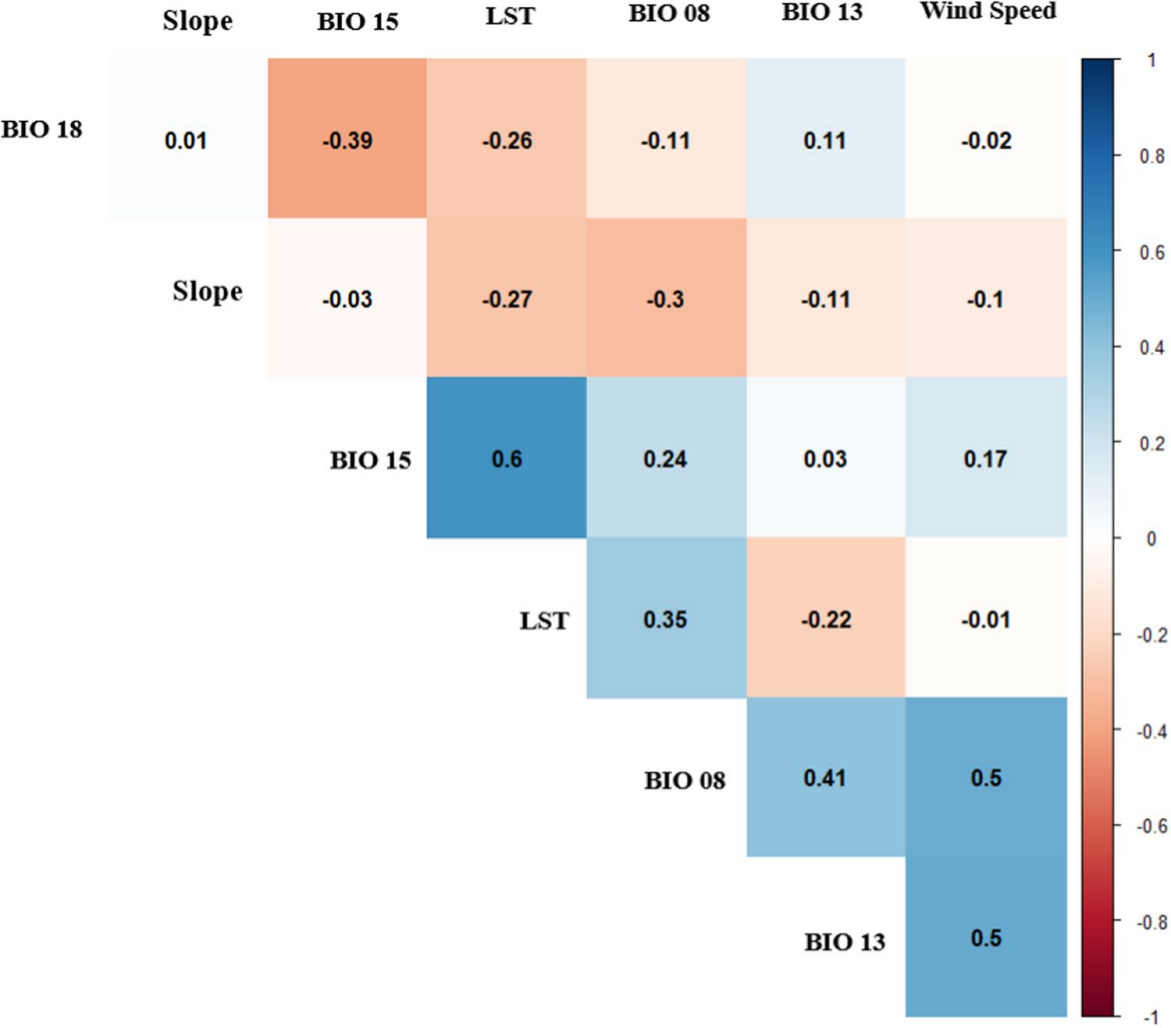
Selection of variables for (ENM) using Spearmans's correlation ( − 0.6 ≤ Α ≤ 0.6). BIO 8 Mean Temperature of Wettest Quarter, BIO13 = Precipitation of Wettest Month, BIO 15 Precipitation Seasonality (Coefficient of Variation), BIO18 = Precipitation of Warmest Quarter, Land Surface Temperature (LST) wind speed and slope

The selection of variables for (ENM) was performed using Spearman's correlation among them, with those showing a correlation ( − 0.6 ≤ *α* ≤ 0.6) being chosen. The selected variables were BIO 8 Mean Temperature of Wettest Quarter, BIO13 = Precipitation of Wettest Month, BIO 15 Precipitation Seasonality (coefficient of variation), and BIO18 = Precipitation of Warmest Quarter, Land Surface Temperature (LST) wind speed and slope (Fig. 1). After applying spatial filters to the occurrence points in RStudio, the data underwent a validation process and were carefully prepared for use in the species distribution modeling (Table 1).

### Performance of the algorithms

**Fig. 2:**
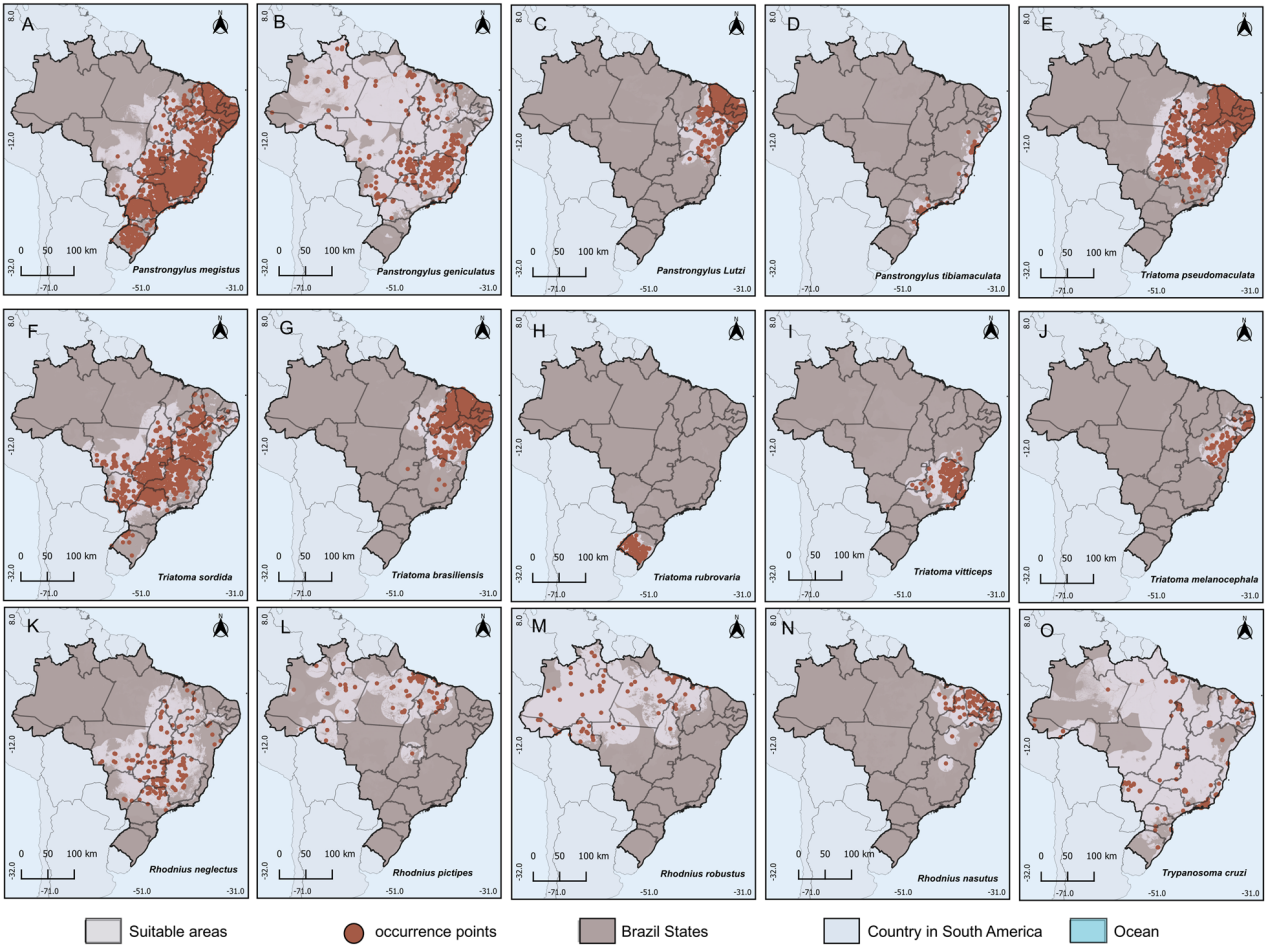
Areas of environmental suitability and occurrence records of triatomines and *Trypanosoma cruzi* in Brazil. Triatomines: **A** *Panstrongylus megistus*, **B** *Panstrongylus geniculatus*, **C** *Panstrongylus lutzi*, **D** *Panstrongylus tibiamaculata*, **E** *Triatoma pseudomaculata*, **F ***Triatoma sordida*, **G ***Triatoma brasiliensis*, **H ***Triatoma rubrovaria*, **I** *Triatoma vitticeps*, **J** *Triatoma melanocephala*, **K** *Rhodnius neglectus*, **L** *Rhodnius pictipes*, **M** *Rhodnius robustus*, **N** *Rhodnius nasutus*, and **O** *Trypanosoma cruzi*

All three models proved effective. RF (Random Forest) and Maxent achieved the highest TSS values for most species, often > 0.90. Although SVM (Support Vector Machine) performed very strongly, it showed slightly greater variation in results in some cases. The similarity in performance among these approaches reinforces the reliability of the projections and suggests that the choice of model may depend on factors such as processing time or the interpretability of the results (Table 2, Fig. 2).

**Table 2:** Predictive performance ranges TSS values for species triatomines and *Trypanosoma cruzi* using three modeling algorithms: Maxent, Random Forest (RF), and Support Vector Machine (SVM)

Triatomines	Maxent	RF	SVM
*Panstrongylus megistus*	0.83–0.87	0.79–0.90	0.79–0.82
*Panstrongylus geniculatus*	0.81–0.90	0.83–0.91	0.78–0.86
*Panstrongylus lutzi*	0.80–0.90	0.87–0.90	0.80–0.89
*Panstrongylus tibiamaculatus*	0.68–0.98	0.76–0.98	0.74–0.98
*Triatoma pseudomaculata*	0.86–0.90	0.87–0.92	0.87–0.90
*Triatoma sordida*	0.85–0.89	0.86–0.92	0.82–0.90
*Triatoma brasiliensis*	0.83–0.91	0.88–0.91	0.87–0.91
*Triatoma rubrovaria*	0.87–0.98	0.87–0.95	0.88–0.91
*Triatoma vitticeps*	0.79–0.93	0.77–0.90	0.71–0.87
*Triatoma melanocephala*	0.81–0.96	0.77–0.95	0.78–0.92
*Rhodnius nasutus*	0.84–0.95	0.76–0.92	0.89–0.95
*Rhodnius neglectus*	0.70–0.89	0.72–0.82	0.73–0.96
*Rhodnius robustus*	0.84–0.99	0.71–0.98	0.91–0.98
*Rhodnius pictipes*	0.77–0.99	0.73–0.99	0.80–0.95
*Trypanosoma cruzi*	0.78–0.89	0.82–0.90	0.71–0.89

Values represent the minimum and maximum TSS scores obtained for each species across cross-validation runs

### Landscape scenarios: a panorama from 1985 to 2023

An analysis of the distribution of 14 species of triatomine bugs from 1985 to 2023 reveals a shift in areas suitable for these insect vectors towards landscapes dominated by human activity. This expansion is primarily driven by agricultural expansion (Additional file 3: Table S1).

**Fig. 3:**
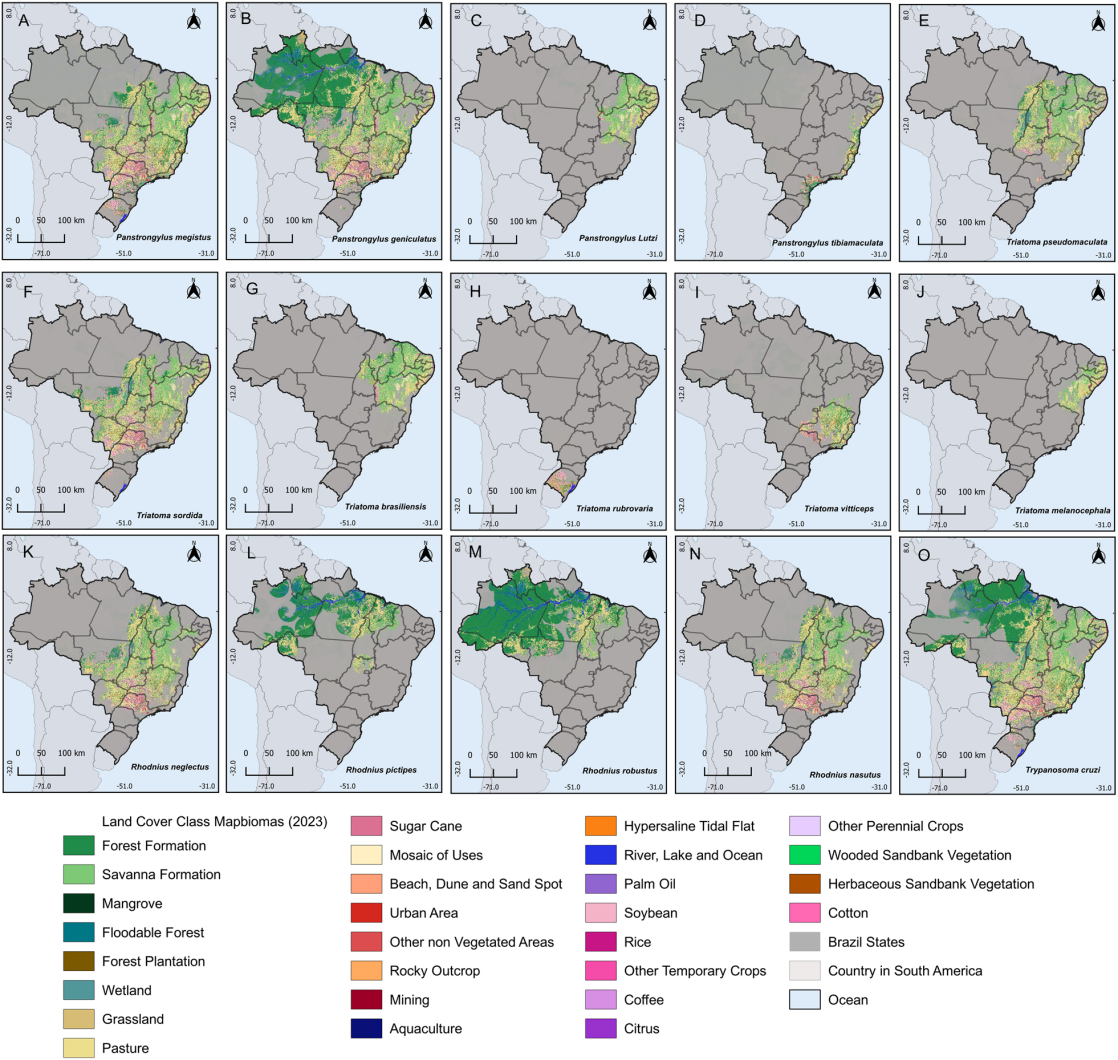
Land use and land cover map in areas of environmental suitability for triatomines and *Trypanosoma cruzi* for the year 2023. Triatomines: **A** *Panstrongylus megistus*, **B** *Panstrongylus geniculatus*, **C** *Panstrongylus lutzi*, **D** *Panstrongylus tibiamaculata*, **E** *Triatoma pseudomaculata*, **F** *Triatoma sordida*, **G** *Triatoma brasiliensis*, **H** *Triatoma rubrovaria*, **I** *Triatoma vitticeps*, **J** *Triatoma melanocephala*, **K** *Rhodnius neglectus*, **L** *Rhodnius pictipes*, **M** *Rhodnius robustus*, **N** *Rhodnius nasutus*, and **O** *Trypanosoma cruzi*

The analyzed species (*Panstrongylus megistus*, *P. geniculatus*, *P. lutzi*, *Triatoma pseudomaculata*, *T. sordida*, *T. brasiliensis*, *T. rubrovaria*, *T. vitticeps*, *T. melanocephala*, *Rhodnius neglectus*, and *R. nasutus*) showed a decrease in occurrence in forest and savannah formations. This loss of natural habitat directly indicates the impact of agricultural expansion on wild vector populations (Fig. 3).

Pasture emerged as the fastest-growing habitat for most species. Specifically, the following species showed the greatest adaptation to this type of landscape: *R. neglectus* (13.06% to 26.33% increase), *T. sordida* (14.45% to 25.17% increase), *T. vitticeps* (13.63% to 24.54% increase), *T. pseudomaculata* (an 8.59% to 24.43% increase), *T. brasiliensis* (5.47% to 23.58% increase), and *R. nasutus* (an 8.43% to 23.78% increase). The expansion of soybean cultivation was also a relevant factor for most species. Their successful adaptation to rural and urban environments suggests a higher risk of transmission to humans.

Though there is a general decline in species association with their original habitats, some species still maintain a strong connection. The results showed that *R. robustus* and *Panstrongylus tibiamaculatus* are mostly found in forest formations (64.69% and 62.78%, respectively), while *Rhodnius pictipes* and *P. geniculatus* are also prevalent in natural environments (Additional file 3: Table S1).

### Spatial distribution and risk classes of triatomines and *Trypanosoma cruzi*

**Fig. 4:**
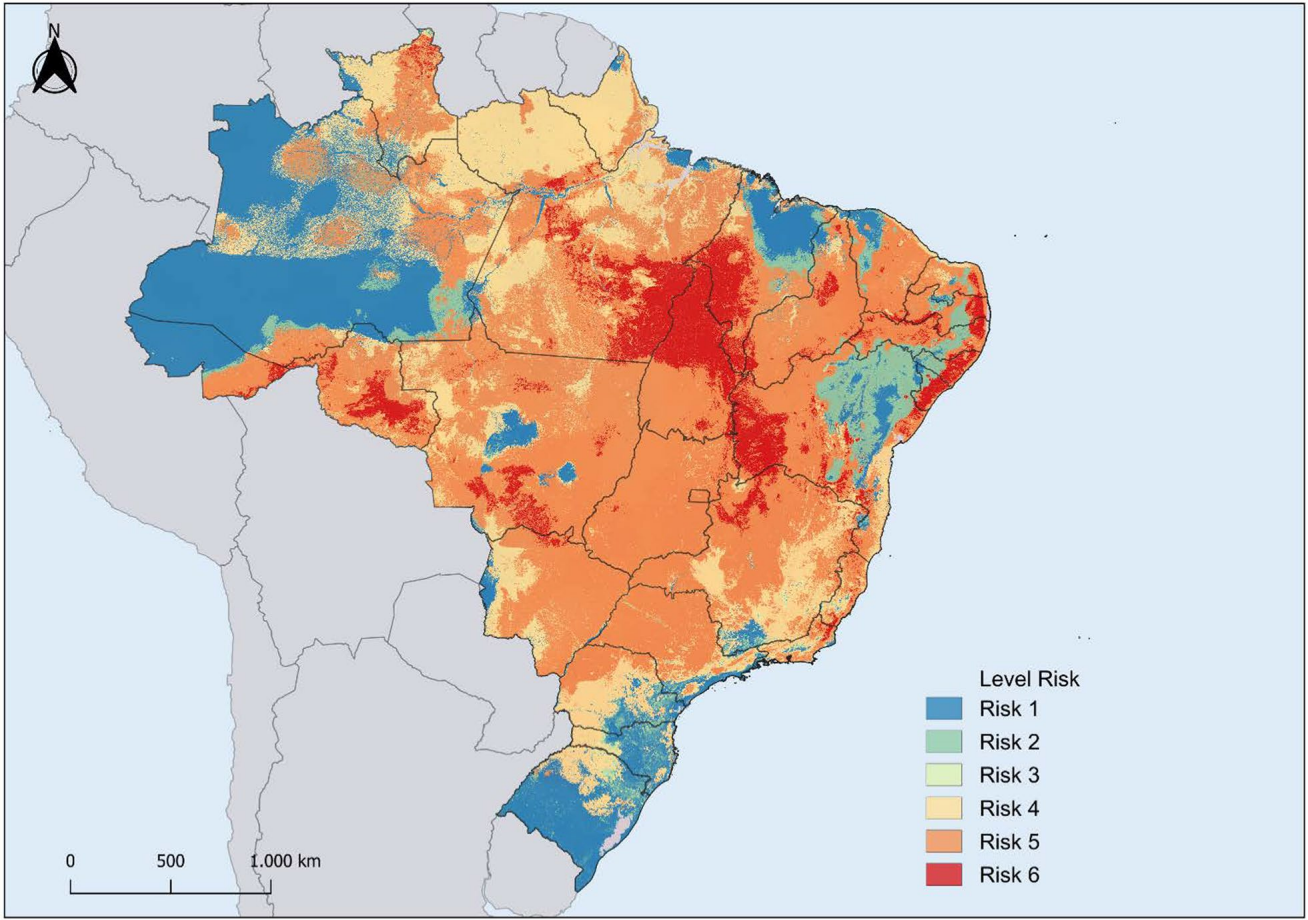
Risk Classification map of *Trypanosoma cruzi* transmission: triatomine species diversity and parasite presence. The risk levels are distributed across the states of Brazil. Dark blue: risk 1; light blue: risk 2; green: risk 3; light yellow: risk 4; orange: risk 5; red: risk 6

The analysis of risk classes by federal unit reveals distinct patterns between regions in Brazil. In the Northern region, there is a high proportion of the highest risk classes in states such as Acre (Class 1: 65%) and Amazonas (Class 1: 59.4%), indicating significant areas at greater risk. On the other hand, states such as Rondônia and Tocantins show a predominance of Class 5 (61.4% and 52.9%, respectively) and Class 6 (19.5% and 46.4%), showing a risk distribution more concentrated in intermediate and high levels. States such as Amapá and Roraima also show a high proportion of Class 4 (81.1% and 42.8%) (Fig. 4).

In the Northeast region, there is great heterogeneity among states. Piauí has a predominance of Class 5 (80.5%), while states such as Alagoas and Sergipe have high proportions of Class 6 (39.4% and 50.5%), indicating greater vulnerability in some locations. States such as Paraíba, Pernambuco, and Bahia have risk distributed among Classes 2, 5, and 6.

In the Southeastern region, the highest risk areas (Class 5) predominate in São Paulo (76.1%) and Minas Gerais (60%), while Rio de Janeiro has a more balanced distribution between Classes 4 and 5 (47.9% and 41.7%, respectively). Espírito Santo follows a similar pattern, with a predominance of Class 4 (36.4%) and 55.1% in Class 5 (Fig. 4).

In the Southern region, there is considerable variation: Rio Grande do Sul shows a predominance of Class 1 (73.3%), while Santa Catarina combines high participation in Classes 1 and 4 (48.6% and 51.2%). Paraná shows a higher concentration in Class 4 (55.1%) and significant participation in Class 1 (14%) (Fig. 4).

In the Midwestern region, Class 5 is dominant in all states, especially in Goiás (96%), Distrito Federal (96.8%), Mato Grosso do Sul (72.3%), and Mato Grosso (74.1%), while Classes 1 and 4 appear only in smaller proportions, suggesting that most of the region is at high risk (Fig. 4).Please write out the names of the Brazilian states or provide a list of the abbreviations.We revised the manuscript and removed the abbreviations of the Brazilian states, replacing them with their full names throughout the text.

These results reflect not only the environmental and land use characteristics of each region, but also the spatial distribution of factors that contribute to greater vulnerability, allowing the identification of priority areas for risk monitoring and mitigation actions (Additional file 4: Table S2).

### Distribution of risk classes by land use and land cover

In the Northern region, forest cover dominates areas of potential risk (Classes 1–3), reflecting the Amazonian matrix. However, in the actual risk classes (5 and 6), there is a greater proportion of pasture areas, especially in Rondônia, Tocantins, and Pará, where forest conversion is strongly associated with increased risk. In Acre and Amazonas, the risk is still concentrated in forest areas, but at higher levels there is also replacement by pastures. Amapá, on the other hand, remains mostly at low risk, with forests still predominating.

In the Northeast, areas of real risk are mainly concentrated in savannahs and pastured, while forest and wetland formations appear to be at lower risk. States such as Maranhão, Rio Grande do Norte, Paraiba Alagoas, and Sergipe show a strong influence of agricultural use and mosaics of use, with areas of greater anthropic pressure associated with high risk.

In the southeast, pastures and savannahs predominate in the real risk classes, reflecting agricultural and urban expansion. In São Paulo, areas of temporary and permanent crops and sugarcane are associated with Class 5, reinforcing the influence of intensive agricultural activity. Minas Gerais, Espírito Santo, and Rio de Janeiro follow a similar pattern, with high risk in anthropic areas. In the midwest, the actual risk is largely associated with pastures, savannahs, and soybeans, while forest areas account for a smaller proportion. Almost the entire territory of Goiás and the Distrito Federal has medium to high risk, and Mato Grosso and Mato Grosso do Sul also show a predominance of agricultural areas in classes 5 and 6, indicating a large area at high risk.

In the south, the risk is relatively lower but still associated with agricultural areas. In Paraná and Rio Grande do Sul, soybeans predominate among the real risk classes, alongside pastures and mosaic land use. In Santa Catarina, urban areas are identified as relevant in Class 5, suggesting risk in scenarios that differ from the region's typical agricultural dynamics (Additional file 5: Table S3).

## Discussion

Previous studies have demonstrated the usefulness of ecological niche modeling for estimating the potential distribution of triatomine vectors in Brazil. Gurgel-Gonçalves [11], modeled the geographic distribution of well-sampled Brazilian triatomine species with synanthropic tendencies at a broad national scale, contributing to the identification of environmentally suitable areas for important Chagas disease vectors. Other studies have also applied similar approaches to specific species or species complexes, such as *Rhodnius neglectus* and *R. nasutus*, as well as the *Triatoma brasiliensis* complex, reinforcing the relevance of ecological modeling for understanding the potential distribution of these vectors [14, 15].

However, the known distribution of triatomines is not static, as new occurrence records, taxonomic updates, environmental changes, and anthropogenic landscape transformations may modify interpretations of suitable areas over time. In this context, the present study advances previous approaches by incorporating an updated occurrence database that includes new distribution areas, and by using environmental and landscape variables that differ from those used in earlier studies. In addition, unlike approaches focused mainly on potential vector distribution, this study integrates suitability models with high-resolution land use and land cover data, enabling assessment of the relationships among suitable areas, landscape transformation, and anthropogenic classes.

The analysis of environmentally suitable areas for 14 triatomine species between 1985 and 2023 reveals a significant ecological transition. Historically, the most suitable areas for these vectors were associated with wild habitats, such as forests and savannahs. However, the main pattern observed is the replacement of these natural ecosystems by agricultural activities, particularly pastures and extensive crops such as soybeans. This land use transformation alters the availability of favorable habitats, forcing vectors to exploit new environments, including areas near human activities, a phenomenon known as peridomestication [16, 17].

Pasture emerged as the land cover category with the greatest expansion, with notable increases in suitability for species such as *R. neglectus* and *T. sordida* (Additional file 1: Fig S1). The spread of soybean cultivation also contributed to the expansion of the environmental suitability of these vectors in rural areas. This pattern suggests that adaptation to altered environments may bring vectors closer to human dwellings, thereby increasing the risk of *T. cruzi* transmission [18].

Although the general trend points to an expansion of suitability in anthropogenic areas, some species maintain high suitability in natural habitats. *Rhodnius robustus* and *P. tibiamaculatus*, for example, still exhibit more than 60% of their environmental suitability within forest formations (Additional file 2: Fig S2). This highlights the importance of preserving natural ecosystems, which are fundamental for restricting the ecological cycle of these species to wild environments and minimizing the risk of peridomestication.

Changes in environmental suitability not only alter the landscape but also bring the *T. cruzi* transmission cycle closer to human populations. Pastures and peri-domestic rural environments provide shelter, blood sources, and domestic hosts, enabling sylvatic vectors to exploit these new niches [19].

This habitat shift has important epidemiological implications. Adaptation of sylvatic triatomines to anthropogenic environments, including pastures, crop fields, and peri-urban areas, increases their interaction with humans and domestic animals [20, 21]. When vectors colonize peri-domestic structures such as chicken coops, corrals, and rural outbuildings, they gain access to abundant food sources, thereby increasing the likelihood of human contact and infection [22].

The risk class analysis revealed distinct vulnerability patterns to Chagas disease across Brazil, with significant variations among the Northern, Northeastern, Midwestern, Southeastern, and Southern regions. These patterns are closely related to the environmental suitability of vectors and the transmission dynamics of *T. cruzi*.

The Northern region showed the highest concentration of acute Chagas disease cases between 2010 and 2019, particularly in Acre, Amazonas, Amapá, Pará, Rondônia, Roraima, and Tocantins. Studies indicate that the predominant transmission route in this region is oral, associated with the consumption of contaminated foods, such as açaí, which attract vectors due to fruit fermentation [23–25]. The expansion of agricultural activities, including the conversion of forests into pastures, has altered natural vector habitats, facilitating proximity to urban areas and increasing transmission risk [24].

In the Northeast, states such as Piauí, Alagoas, and Sergipe showed a high proportion of areas classified as high real risk (Classes 5 and 6). Agricultural expansion and urbanization have converted natural formations into cropland and pasture, thereby favoring the presence of triatomines and parasite transmission [25]. The prevalence of chronic Chagas disease remains high, reflecting the disease's persistence in the region [26].

The Midwestern region, comprising Goiás, Mato Grosso, and Mato Grosso do Sul, had a high incidence of Chagas disease, especially in Goiás. Pasture and soybean cultivation dominate the landscape, favoring vector adaptation to modified environments [27]. Mortality trends in the region have shown variation, with reductions in some states, suggesting improvements in healthcare infrastructure and services.

In the southeast, states like São Paulo and Minas Gerais presented areas of high real risk associated with intensive agriculture, such as sugarcane and soybean cultivation. Conversion of natural habitats into crop areas has increased the presence of triatomines and *T. cruzi* transmission [18]. Effective surveillance and control are crucial to prevent outbreaks in this region [28].

The south exhibited generally lower risk for Chagas disease transmission, with a predominance of low-risk areas (Classes 1 and 2). However, the presence of high real-risk areas associated with pastures and soybean crops indicates that landscape modification can still favor triatomine occurrence, even in historically less endemic regions [29, 30]. Continuous monitoring is essential to detect potential changes in transmission dynamics [28]. 

Understanding these spatial patterns, together with climatic and landscape characteristics, is essential for interpreting the ecology of *T. cruzi* transmission and identifying areas where vector surveillance should be intensified [31]. In landscapes dominated by soybean cultivation and pasture, especially where these land use classes overlap with areas of high suitability for triatomine vectors, the maps may also guide health education activities directed at rural residents, agricultural workers, community health agents, and local surveillance teams. The maps generated in this study may support the planning and implementation of more targeted Chagas disease surveillance strategies [32], Identifying the main triatomine species expected in each region, these maps can support more targeted educational materials, including species-specific images, information on domestic and peridomestic ecotopes, guidance on safe collection, and instructions on how to deliver suspected insects to Triatomine Information Posts (TIPs). They may also help prioritize municipalities and localities for the establishment or strengthening of TIPs.[33]. Areas classified as having higher environmental suitability or higher risk may be used to guide active entomological surveillance, household and peridomestic inspections, and community-based notification systems. This approach is consistent with Brazilian vector surveillance strategies, in which residents are encouraged to collect suspected triatomines and deliver them to TIPs, and with evidence that community participation contributes to long-term Chagas disease vector surveillance [34, 35]. In addition, educational materials focused on the predominant local triatomine species can facilitate species recognition by field teams and the population, supporting surveillance actions, training activities, and citizen-based notification of suspected triatomines [36].Please check the edits in '...and instructions on delivering suspected insects to Triatomine Information Posts (TIPs). They particularly help to prioritize municipalities and localities for the establishment or strengthening of TIPs'.We checked the edited sentence and made a minor adjustment to improve clarity. The revised text now reads: “instructions on how to deliver suspected insects to Triatomine Information Posts (TIPs).”In figures 2 and 3 part labels K was missing, hence were have changed, kindly check and confirm.We revised the manuscript accordingly, and the corrected version has been included in the revised submission and supplementary attachments

Although the present study provides useful information for identifying environmentally suitable areas for triatomine vectors, some limitations should be considered. Occurrence records may also be affected by spatial sampling bias and spatial autocorrelation, especially in areas with greater sampling effort, which can influence model calibration and inflate model evaluation metrics [37–39]. To reduce these effects, we adopted detailed data curation and spatial filtering procedures, an approach commonly used to reduce spatial redundancy and overfitting to biased sampling patterns in ecological niche models [40, 41]. Nevertheless, such bias may not be eliminated in occurrence-based ecological niche models, and the suitability maps should therefore be interpreted as estimates of environmental suitability rather than absolute species distributions [39–42].

For this reason, human population density was not included as a predictor in the models. Although this variable may be important for estimating human exposure and transmission risk, human infection by *T. cruzi* is shaped by ecological, socioeconomic, and cultural factors operating at different spatial scales [43, 44]. However, human population density does not necessarily represent the availability of suitable habitats or ecotopes for triatomines, especially for species strongly associated with sylvatic and peridomestic environments [11–45]. In this study, anthropogenic influence was represented by land use and land cover variables that capture landscape modification, habitat structure, and the extent of environmental transformation. Previous studies have shown that anthropogenic land use change, deforestation, forest remnants, and landscape transformation can influence triatomine abundance, infestation patterns, and vector-human contact [22–47].

In addition, land use change is a continuous process, and achieving a perfect temporal match between entomological records and landscape conditions is difficult when using historical occurrence databases. Thus, the models should be interpreted using the best available landscape information for the analyzed period. This limitation is consistent with broader discussions in species distribution modeling, which emphasize that temporal mismatch between occurrence records and dynamic environmental predictors can affect model interpretation and that temporally explicit predictors may improve future models [48].

Triatomine occurrence is not determined exclusively by landscape structure and environmental suitability; ecological interactions, particularly the availability and diversity of vertebrate hosts, may also influence vector presence, abundance, and infection patterns [49, 50]. Mammalian reservoirs can contribute to the maintenance of enzootic transmission cycles by providing blood-meal sources and sustaining *Trypanosoma cruzi* circulation in natural and peridomestic environments [6, 51, 52]. We clarified that the maps should be interpreted as estimates of environmental suitability and surveillance priority rather than as absolute predictions of vector distribution or human infection risk. Future studies integrating landscape predictors, mammal reservoir distribution, host community composition, vector infection data, human population density, housing conditions, and socioeconomic indicators may provide a more comprehensive assessment of the risk of Chagas disease transmission.

## Conclusions

Our conclusions highlight the association between human-modified landscapes, such as agricultural areas, and the presence of *T. cruzi* and its vectors. This underscores the critical role of these landscapes in shaping transmission risk. Continuous monitoring of these environments is essential to minimize the likelihood of outbreaks and new cases of Chagas disease. The spatial distribution of risk classes revealed areas at higher risk of transmission, providing valuable guidance for targeted surveillance and control efforts. These results highlight the importance of considering both landscape characteristics and anthropogenic pressures when designing integrated disease prevention strategies. Aligning ecological insights with public health interventions strengthens surveillance systems and supports the development of more effective, sustainable control policies for Chagas disease.

## Supplementary Information

Additional file 1. Figure S1: The distribution of land use and land cover classes within the suitability zones is shown for (A) *Rhodnius neglectus* and (B) *Triatoma sordida* in Brazil. The maps use the following color scheme to illustrate landscape dynamics: remaining forest (light green), regeneration forest (dark green), anthropized areas (yellow), and deforestation (red). For the species *R. neglectus* and *T. sordida*, the niche suitability shows a strong presence of yellow (anthropized areas) and red (deforestation), particularly in the central regions of Brazil (the Cerrado biome), indicating that these species maintain high suitability in environments heavily modified by human activity.

Additional file 2. Figure S2: The distribution of land use and land cover classes within the suitability zones is shown for *Rhodnius robustus* (A) and *Panstrongylus tibiamaculatus* (B) in Brazil. The maps use the following color scheme to illustrate landscape dynamics: remaining forest (light green), regeneration forest (dark green), anthropized areas (yellow), and deforestation (red). The *R. robustus* map is dominated by light green, reflecting its primary association with the Amazon biome and remaining forest. However, significant red and yellow patches (deforestation and anthropization, respectively) are visible, indicating that these environmental changes are encroaching upon the species natural habitat. *Panstrongylus tibiamaculatus*: this species shows a niche restricted to the Atlantic Forest coast, where there is a high fragmentation of colors. While light green (remaining forest) persists in mountainous or protected areas, there is a high density of yellow (anthropized) and dark green (regeneration forest) pixels, highlighting the species' presence in a highly modified and recovering landscape.

Additional file 3. Table S1: Percentage distribution of triatomine species occurrences by land use and land cover category (1985-2023). This table shows how the occurrence of different triatomine species was distributed across landscape types in two time periods, revealing the ecological transition of the vectors.

Additional file 4. Table S2: Risk classification of *Trypanosoma cruzi* transmission in Brazil: triatomine species diversity and *T. cruzi* occurrence.

Additional file 5. Table S3: Land use and land cover category (1985–2023) in risk classification areas of *Trypanosoma cruzi* transmission in Brazil

## Data Availability

Data supporting the main conclusions of this study are included in the manuscript.
